# Investigations of Effects of Radiotherapy, Sonic Activation and Root Canal Treatment on Fracture Resistance of Mandibular Anterior Teeth: An In Vitro Study

**DOI:** 10.3390/jcm15052066

**Published:** 2026-03-09

**Authors:** Fatma Tunc, Nihat Sahin, Ihsan Karslioglu, Sule Baz Cifci, Mustafa Ozgul

**Affiliations:** 1 Department of Endodontics, Faculty of Dentistry, Gaziantep University, Gaziantep 27310, Türkiye; 2 Department of Radiation Oncology, Faculty of Medicine, Gaziantep University, Gaziantep 27310, Türkiye; 3 Department of Ophthalmology, School of Medicine, University of California Irvine, Irvine, CA 92617, USA

**Keywords:** endodontics, fracture resistance, radiotherapy, root canal treatment, sonication

## Abstract

**Background and objectives**: Head and neck cancer patients frequently undergo radiotherapy, which can affect the properties of dental hard tissues. This study aimed to evaluate the effects of root canal treatment, radiotherapy, and sonic activation during irrigation on the fracture resistance of mandibular anterior teeth. **Methods**: 80 extracted mandibular anterior teeth were randomly divided into five groups: untreated control (Group I); root canal treatment without radiotherapy or sonic activation (Group II); root canal treatment without radiotherapy but with sonic activation (Group III); root canal treatment with 70 Gray (Gy) radiotherapy and sonic activation (Group IV); and root canal treatment with radiotherapy but without sonic activation (Group V). Radiotherapy was administered in fractionated doses (2 Gy/day, 5 days/week) over 7 weeks. Following instrumentation, root canal obturation was performed accordingly. Fracture resistance was measured using a universal testing apparatus with vertical loading until fracture. Statistical analyses included Shapiro–Wilk normality testing followed by appropriate non-parametric Kruskal–Wallis test followed by Dunn’s post hoc test with Bonferroni correction for multiple comparisons. **Results**: All root canal-treated groups exhibited significantly lower fracture resistance compared to the untreated control group [1572.3 (1217.0–1841.2) N, *p* < 0.05]. No statistically significant differences were observed between irradiated and non-irradiated groups (*p* > 0.05). Similarly, sonic activation during irrigation did not significantly affect the fracture resistance values (*p* > 0.05). **Conclusions**: Under the specific conditions of this in vitro protocol, fractionated radiotherapy and sonic activation did not demonstrate statistically significant effects on fracture resistance in mandibular anterior teeth, while endodontic procedures reduced fracture resistance.

## 1. Introduction

For over a century, radiotherapy has revolutionized cancer treatment and is one of the fundamental treatments in head and neck cancers [1,2,3]. The primary objective of radiotherapy is the delivery of a cytotoxic dose of ionizing radiation to malignant cells to eradicate the cancer cells [4]. Technological advancements have aimed to deliver high doses to tumors with increased precision while minimizing off-target effects. However, the anatomical architecture of the head and neck region has multiple critical structures (teeth, salivary glands, alveolar bone) within or in close proximity to the radiation field. Preventing the radiation-induced damage in these healthy tissues remains a significant challenge that can compromise treatment outcomes and patient quality of life [4].

Radiation exposure affects the mechanical properties of dental hard tissues. The specific effects of radiation are dose-dependent in teeth [5,6,7]. Dentin exhibits greater vulnerability, with microhardness decreasing after 10, 20, 30, 50, and 60 Gray (Gy) cumulative doses, particularly in the middle segment, whereas superficial and deep dentin show minimal alterations [5]. These findings emphasize the susceptibility of dentin to radiation-induced structural changes, which may directly influence the biomechanical behavior of the middle root region [5].

These radiation-induced changes extend to the microstructure of dental tissues. After 30–60 Gy exposure, obliterated dentinal tubules and progressive fragmentation of collagen fibers were demonstrated using scanning electron microscopy (SEM) analysis [5]. A previous study showed significant decreases in microhardness and elastic modulus near the dentinoenamel junction in irradiated teeth [6]. These changes could negatively affect the fracture resistance of irradiated dental tissues. A previous study demonstrated that both radiotherapy timing and material selection influenced the biomechanical properties of dental tissues [8].

Root canal treatment aims primarily to eliminate apical periodontitis and prevent its recurrence. This requires effective removal of bacterial biofilm, bacteria, necrotic pulp tissue and toxins from the root canal system [9,10]. Due to the narrow dimension of root canals and limitations of conventional irrigation, sonic activation methods have been developed to enhance the effectiveness of irrigation procedures [10].

Sonic activation is shown to help remove debris and smear layer [11,12]. This technique can reach areas that are difficult to clean efficiently [12]. Such activation generates fluid dynamics that interact with tooth structures [10,13]. Compared to higher-frequency ultrasonic systems, this method produces less dentinal damage [10,14]. The lower operating frequency results in reduced stress concentration on canal walls [10,13]. Overall, this activation procedure represents effective canal cleaning with preservation of tooth structure [10,12,13].

Previous studies have evaluated the effects of various activation methods and radiation exposure on tooth structures [5,6,7,8,10,11,12,13,14,15]. However, the findings remain inconclusive and inconsistent. While some studies have shown that exposure to 60 Gy of radiation does not significantly affect dentin microhardness [16] or the fracture resistance of teeth [8], other investigations have reported that radiotherapy results in a decrease in both dentin hardness values and fracture strength [5,15]. Activation methods in endodontics include sonic activation (operating at lower frequencies of 1–10 Kilohertz (kHz)) and ultrasonic activation (25–30 kHz). These systems work through different mechanisms: ultrasonic activation relies on cavitation and acoustic streaming, while the sonic system operates through mechanical agitation at lower frequencies [17,18].

Likewise, the available evidence on the effects of irrigant activation methods on dentin microhardness and fracture resistance remains conflicting [19,20]. Whereas the effects of sonic activation on root dentin ultrastructure, especially microhardness and cavitation, have been documented [21,22], there is limited evidence regarding its effect on root fracture resistance [23]. However, the combined impact of sonic activation and high-dose radiotherapy (70 Gy) on the fracture resistance of intact teeth and endodontically treated teeth has not been thoroughly investigated. Fracture strength represents a clinical parameter for assessing the long-term prognosis of teeth, particularly in patients who have received radiotherapy for head and neck malignancies. For head and neck cancer survivors who require endodontic treatment, investigating these potential effects on fracture resistance could contribute to our understanding that may help minimize tooth loss and maintain oral function.

Timing of radiotherapy and root canal treatment depends on multiple factors including stage, tumor growth rate, type of head and neck cancer, patient prognosis, and treatment urgency. More than half of head and neck oncology patients are diagnosed at advanced stages [24]. Head and neck malignancies are generally aggressive with limited time intervals between treatment decisions and radiotherapy initiation [25]. When head and neck cancer demonstrates rapid progression or patients present with advanced disease, radiation oncologists may determine that radiotherapy must proceed without delay, even before completion of recommended dental procedures. Additionally, cancer survivors who have completed radiotherapy may develop radiation-induced endodontic pathology, requiring post-irradiation root canal treatment [26].

We hypothesize that there is no significant difference in fracture resistance between untreated teeth and teeth that have undergone root canal treatment with specific protocols (with/without radiotherapy, with/without sonic activation). This study aimed to evaluate the effects of root canal treatment, radiotherapy, and sonic activation during irrigation on the fracture strength of mandibular anterior teeth.

## 2. Materials and Methods

### 2.1. Ethics Approvals

This laboratory investigation was conducted in accordance with the ethical principles of the Declaration of Helsinki and its later amendments. The study protocol was reviewed and approved by the Ethics Committee of Gaziantep University (approval number: 2022/338). All specimens were collected after obtaining written informed consent from patients.

### 2.2. Sample Collection and Preparation

The present study focused on mandibular anterior teeth with single and straight canals that were extracted from dental patients aged 30–45 for periodontal or orthodontic reasons. Initially, the mesio-distal and bucco-lingual dimensions of the obtained roots in the coronal region were measured using a digital caliper (Digimess, São Paulo, Brazil), and roots with similar dimensions were selected for the study. The teeth were analyzed using microscopy for cracks, followed by digital periapical radiographs to confirm single-canal anatomy. Surface residues were cleaned with a brush and a 0.1% thymol solution was used for storage and disinfection.

**Figure 1:**
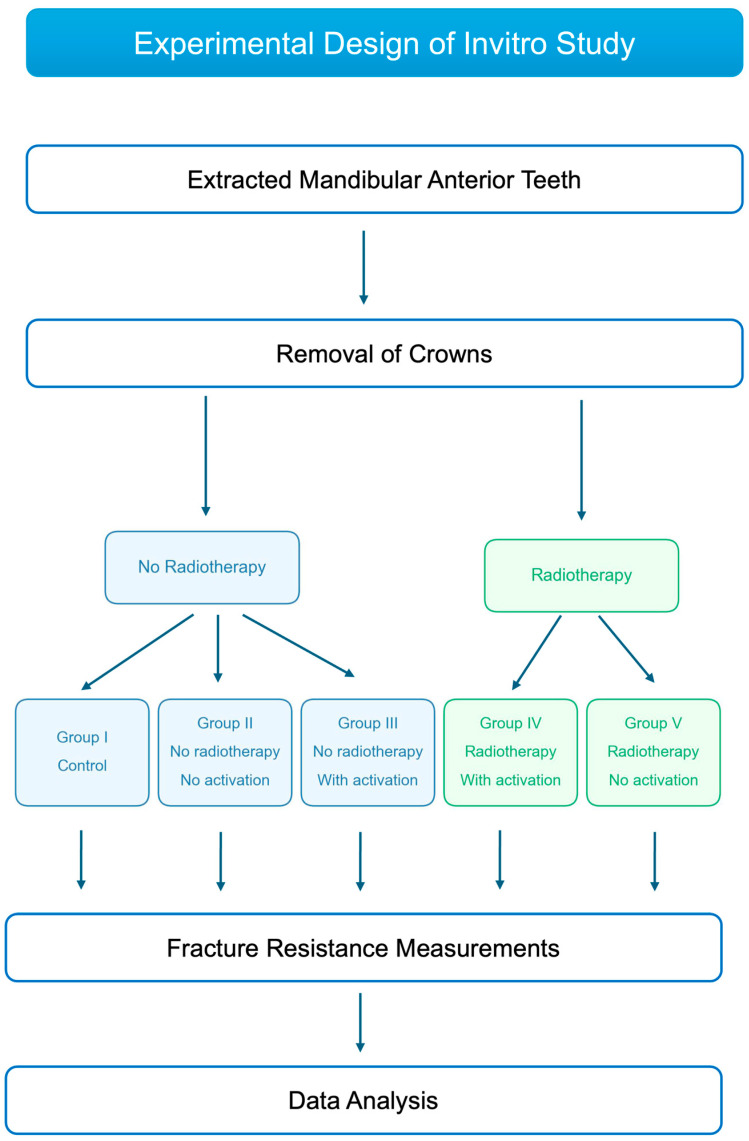
Experimental workflow for investigating the effects of radiotherapy and sonic activation on fracture resistance of endodontically treated teeth. All groups underwent fracture resistance measurements followed by statistical data analysis. The color-coding shows teeth samples with radiotherapy (green) and without radiotherapy (blue).

For standardization, all teeth were sectioned to 13 mm (mm) from apical to coronal ends [27], with crowns removed using a fissure bur under water cooling. The digital caliper was used to measure buccolingual and mesiodistal dimensions at the cutting surface, and all measurements were recorded in millimeters (mm). To maintain standardization, teeth were included only when their dimensions were within 10% of the calculated dimensional mean [28]. The 80 teeth samples were selected and randomly divided into five groups according to the treatment protocols as seen in Table 1 and Figure 1. Root canal treatment was performed on all experimental groups except the control group.

### 2.3. Root Canal Preparation Protocol

The initial working length was determined by using a size 10 K-file (VDW, Munich, Germany), which was inserted until its tip became visible at the apical foramen and then withdrawn 1 mm. The root canals were initially instrumented with a size 15 K-file and subsequently with rotary files up to 35.04 (Race EVO, FKG Dentaire SA, La Chaux-de-Fonds, Switzerland). The irrigation was conducted with 1 mL of 2.5% NaOCl between each file, administered through a 30-gauge double-side-vented needle (Navitip; Ultradent Products, South Jordan, UT, USA).

The experimental design included the following five groups:

Group I (Control, *n* = 16): No treatment was performed on these teeth samples.

Group II (*n* = 16): First, root canal preparation was performed with rotary files up to size 35.04. Then, conventional irrigation was applied without sonic activation.

Group III (*n* = 16): First, root canal preparation was performed with rotary files up to size 35.04. Then, conventional irrigation was applied followed by sonic activation.

Group IV (*n* = 16): First, radiotherapy (70 Gy) was administered. Then, root canal preparation was performed with rotary files up to size 35.04. After that, conventional irrigation was applied followed by sonic activation.

Group V (*n* = 16): First, radiotherapy (70 Gy) was administered. Then, root canal preparation was performed with rotary files up to size 35.04. After that, conventional irrigation was applied without sonic activation.

### 2.4. Final Irrigation Protocol

The final irrigation protocols were as follows: For Groups II and V (without sonic activation), the root canals were irrigated with 10 mL of 2.5% NaOCl for 1 min, followed by 5 mL of 17% ethylenediaminetetraacetic acid (EDTA), and a final rinse with 10 mL of distilled water. For Groups III and IV (with sonic activation), the root canals were first irrigated with 10 mL 2.5% NaOCl, which was then agitated with an EndoActivator (Dentsply, Tulsa, OK, USA) handpiece set at 10,000 cycles per minute using a red (25/04) tip placed at a point 2 mm short of the working length for 1 min (activation time). Following sonic activation, these canals were irrigated with a 5 mL 17% EDTA solution to remove the smear layer and flushed with 10 mL distilled water.

### 2.5. Obturation Protocol

All treated canals were obturated with a 35/04 gutta-percha master cone and Endoplus sealer (President Dental, Duisburg, Germany). The samples were preserved at 100% relative humidity and at room temperature until testing for fracture resistance.

### 2.6. Radiotherapy Protocol

Teeth samples in Groups IV and V were placed in plastic vials containing distilled water to receive a uniform radiation dosage. A cumulative dose of 70 Gy was chosen to mimic standard clinical regimens for advanced-stage head and neck cancer patients, as recommended in clinical guidelines [29] and previously applied in dental radiotherapy studies [30,31]. This protocol delivered a total dose of 70 Gy in 35 fractions (2 Gy/day, 5 days/week) over a period of 7 weeks using a radiotherapy device (Varian Clinac DBX 600, Varian Medical Systems, Palo Alto, CA, USA). Preservation of roots in distilled water was ensured both before and after radiotherapy for renewal. The irradiation was performed at the Department of Radiation Oncology, Faculty of Medicine, Gaziantep University in Gaziantep, Turkiye.

### 2.7. Fracture Resistance Testing

For the simulation of periodontal ligament, the apical 10 mm of each root was coated with a wax layer of uniform thickness (0.2–0.3 mm) [32] and measured using a digital caliper. The teeth samples were embedded vertically to a depth of 10 mm in self-curing resin cylinders (length 15 mm × width 20 mm). With the initiation of polymerization of the acrylic resin, the roots were separated from the resin, and the wax was removed. After being reinserted into the acrylic resin, the root surfaces were coated with a slim stratum of polyvinylsiloxane impression material (Ultradent Products, Inc., South Jordan, UT, USA). A universal testing apparatus (Shimadzu Autograph AGS-X, Tokyo, Japan) was used to measure fracture resistance. The instrument’s lower plate held the acrylic blocks in place, whereas the upper plate was fixed with a steel tip in conical form. The tip crushed the canal’s center and applied a vertical load of one millimeter per minute until the tooth fractured. Each root’s maximum fracture force was recorded in Newtons (N).

### 2.8. Statistical Analysis

Statistical analyses were performed using Project Jupyter with Python (version 3.12.4) and the NumPy (version 1.26.4), Pandas (version 2.3.1), SciPy (version 1.11.4), Matplotlib (version 3.10.0), Seaborn (version 0.13.2), and statsmodels libraries (version 0.14.4), as well as SPSS (version 22.0) for tooth dimension analyses.

The normality of fracture resistance and tooth dimension (buccolingual and mesiodistal) data was assessed using the Shapiro–Wilk test. Buccolingual and mesiodistal measurements were normally distributed and were compared among groups using one-way analysis of variance (ANOVA). Descriptive statistics were expressed as mean ± standard deviation (SD).

Normality was assessed using the Shapiro–Wilk test. As not all groups followed a normal distribution (*p* < 0.05 for Group II), intergroup differences were analyzed using the Kruskal–Wallis H test (H = 24.14, *p* < 0.0001) followed by Dunn’s post hoc test with Bonferroni correction for multiple comparisons.

**Figure 2:**
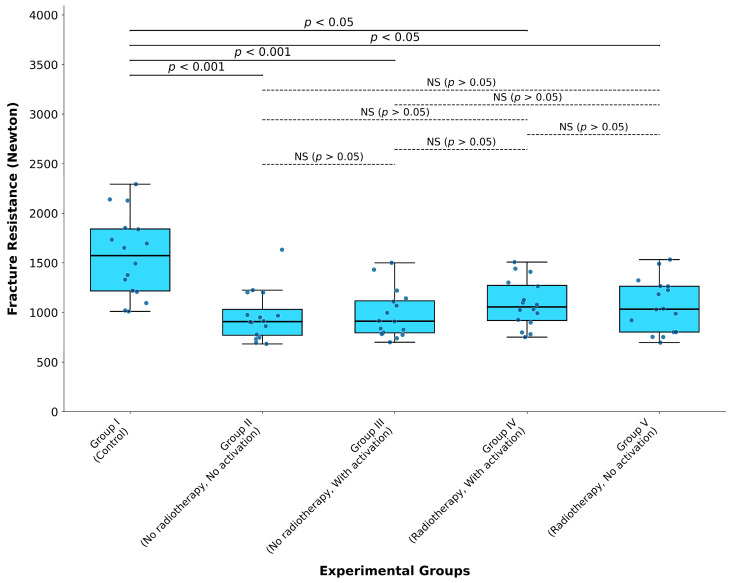
Box-and-whisker plot showing the median fracture resistance values (in Newtons) for mandibular anterior roots across the five experimental groups. Boxes represent the interquartile ranges (Q1–Q3), with the horizontal line indicating the median. Whisker extend to 1.5x IQR. Individual data points are displayed as small blue circles overlaid on each box. Solid lines with corresponding *p*-values indicate statistically significant differences between groups (*p* < 0.001 or *p* < 0.05), whereas dashed lines labeled “NS” denote non-significant differences (*p* > 0.05) between groups.

Statistical significance was established at *p* < 0.05, with additional significance thresholds at *p* < 0.01 and *p* < 0.001 for stronger effects. Fracture resistance results were presented as medians with interquartile ranges (IQRs) in box-and-whisker plots with individual data points overlaid to illustrate distribution of data points. Statistical significance between groups was indicated graphically using lines with corresponding *p*-values as shown in Figure 2. Non-significant comparisons (*p* > 0.05) were denoted as “NS, non-significant” with dashed connecting lines.

### 2.9. Power Analysis

Sample sizes were determined based on a similar study investigating radiotherapy effects on tooth fracture resistance [8]. Post hoc power analysis showed statistical power (1-β) for comparisons between the control group and treatment groups (power: 0.87–0.98). These comparisons demonstrated large effect sizes (Cohen’s d: 1.42–1.78). The current sample sizes (*n* = 16 per group) exceeded the requirements for achieving 80% power.

## 3. Results

### 3.1. Analysis of Anatomic Dimensions

Baseline buccolingual and mesiodistal root dimensions were comparable among all experimental groups, with no statistically significant differences detected (*p* > 0.05), indicating adequate group homogeneity. Descriptive statistics (mean ± SD) of measurements are summarized in Table 2.

### 3.2. Fracture Resistance Analysis and Statistical Comparison

The Kruskal–Wallis H test revealed significant differences in fracture resistance among the experimental groups (H = 24.14, *p* < 0.0001). Figure 2 presents the statistical comparisons of fracture resistance values across all experimental groups.

In Figure 2, statistical analysis revealed that the control group [1572.3 (1217.0–1841.2) N] demonstrated significantly higher fracture resistance compared to Group II [908.4 (769.8–1031.3) N] (*p* < 0.001) and Group III [912.1 (794.2–1117.1) N] (*p* < 0.001). Significant differences were observed between the control group and Group IV [1054.9 (919.1–1272.4) N] (*p* < 0.05) and Group V [1033.6 (802.0–1264.0) N] (*p* < 0.05).

Group II and Group III showed no statistically significant difference (*p* > 0.05), as summarized in Figure 2. Group II did not exhibit significantly lower fracture resistance than Group IV (*p* > 0.05). Similarly, Group II indicated no significantly lower values compared to Group V (*p* > 0.05).

Figure 2 illustrates that there were no statistically significant differences among Group III and the radiotherapy groups (IV and V) (*p* > 0.05), nor between Group IV and Group V (*p* > 0.05).

## 4. Discussion

The present research investigated the effects of root canal treatment, radiotherapy, and sonic activation on the fracture resistance of mandibular anterior teeth in a laboratory setting. To our knowledge, this is the first study to investigate the combined effects of high-dose radiotherapy (70 Gy) and sonic activation on the fracture resistance of intact and endodontically treated teeth.

Our study demonstrated that root canal treatment significantly reduced fracture resistance compared to untreated controls, regardless of additional protocols involving radiotherapy or sonic activation. These findings align with previous studies that show endodontic procedures weaken fracture strength values compared to untreated controls [33,34]. Our measurements align with Alkahtany research (2021), which found that untreated teeth have nearly 1.5 times the fracture resistance of teeth with root canal treatment [35]. These results emphasize the importance of understanding mechanisms that decrease the fracture resistance in teeth after endodontic procedures.

Radiotherapy is one of the primary curative-intent treatment modalities for head and neck cancers at both early and advanced stages, with doses ranging from 54 to 70 Gy delivered using a conventional fractionation schedule of 2 Gy/fraction, 1 fraction per day, 5 fractions per week [29]. Our study utilized a high-dose protocol of 70 Gy delivered in 35 fractions (2 Gy/day, 5 days/week over 7 weeks) to simulate clinical treatment regimens used for advanced-stage head and neck cancer patients. This radiation protocol was selected based on its clinical relevance and consistency with previous dental radiotherapy research [31].

The current findings revealed no statistically significant differences in fracture resistance between irradiated and non-irradiated anterior mandibular teeth after delivering a total dose of 70 Gy. These results align with aspects of Lu et al., who used a similar fractionated protocol (2 Gy/fraction/day, 5 days/week) to achieve total doses of 30 Gy and 60 Gy on extracted non-carious third molars [6]. Lu et al. study demonstrated that there were no significant differences in microhardness or elastic modulus values among the groups in middle dentin, middle enamel, and 50 μm from the dentinoenamel junction [6]. These findings suggest that when using clinically relevant fractionated protocols, certain mechanical properties of specific regions of dental tissues may maintain their integrity following radiotherapy. This consistency in findings across different tooth types (anterior mandibular teeth versus third molars) contributes to understanding the radiobiological effects on dental structures.

Our results showed no significant differences in fracture resistance between groups with and without radiotherapy when using gutta-percha with Endoplus sealer, aligning with the gutta-percha/sealer findings from a previous research study that investigated the effect of radiotherapy timing and obturation materials on fracture resistance [8]. This previous study demonstrated that for teeth filled with gutta-percha/sealer, they found no significant difference in fracture resistance whether radiotherapy was applied before or after obturation [8]. However, teeth filled with Biodentine revealed significantly lower fracture resistance when radiotherapy was applied after obturation in that study. These in vitro study findings highlight the importance of material selection and radiotherapy timing in endodontic treatment protocols.

Previous research has revealed important mechanical alterations following radiotherapy [7]. Goncalves et al. demonstrated that while overall enamel microhardness remained relatively unchanged at higher radiation doses (40–60 Gy), superficial enamel microhardness increased significantly at 40, 50, and 60 Gy cumulative doses [5]. Dentin structure exhibited significant decreases in microhardness after specific radiation doses (10, 20, 30, 50, and 60 Gy cumulative doses) in the middle of the dentin (between the superficial layer near the dentinoenamel junction and the deep layer near the pulp chamber) [5].

The present outcomes contrast with Soares et al., who reported that gamma irradiation therapy (60 Gy in daily increments of 2 Gy) significantly reduced the fracture resistance of intact premolar teeth [15]. The different findings between our study and those of Soares et al. and Goncalves et al. may be attributed to variations in experiment design, tooth type, or the specific mechanical parameters being measured [5,15]. In their study, Soares et al. performed a vertical fracture test on the maxillary premolar teeth without separating them from their crowns, whereas in our study, the lower anterior teeth were separated from the crown parts and only root dentin was used [15]. The possibility of different levels of impact of ionizing radiation on enamel and dentin may be cited as the reason for the difference in study results. Cunha et al. have already examined three different regions of extracted teeth, from cervical to occlusal, and observed reduced microhardness only in the cervical region [30]. Considering the occurrence of radiation caries mostly in the cervical part of the crowns, the stimulation of the initial fracture against vertical forces may be attributed to stress accumulation near the enamel-dentin junction. Furthermore, the inclusion of amalgam and resin restorations in the study by Soares et al. introduces an additional variable that may have influenced fracture strength outcomes, considering the differential response of dental hard tissues and restorative materials to ionizing radiation. These contrasting results highlight the importance of various radiation effects on dental tissues.

The presence or absence of sonic activation did not result in statistically significant differences in fracture resistance values. These results align with a previous study that sonic-assisted method did not affect microhardness measurements [21]. Furthermore, our findings are directly corroborated by Abdulrahman and Faraj, whose comparative analysis demonstrate that there is no meaningful difference in fracture strength values between conventional syringe irrigation (with NaOCl and EDTA) and sonic-activated irrigation with EndoActivator [23]. These consistent findings across different mechanical properties suggest that activated irrigation protocols do not substantially alter the structural integrity of endodontically treated teeth compared to conventional irrigation methods.

Cleaning solutions used for irrigation during root canal treatment can alter the chemical compound by dissolving the inorganic and organic structures of root dentin. Sodium hypochlorite, which has antibacterial properties and is frequently used in root canal irrigation, has a proteolytic effect, whereas EDTA, a chelating agent, causes decalcification in inorganic tissue [36,37]. Some studies incorporating different irrigation protocols have demonstrated an impact on dentin microhardness [38,39]. Chemical-induced changes, including the dissolution of both organic and inorganic components of dentin, cause a decrease in the elastic modulus and fracture strength of dentin, making root canal-treated teeth more susceptible to damage from chewing forces [40]. A recent review emphasized that the effects of irrigants on dentin structure vary depending on application time and concentration [41]. For this reason, the same concentration and amount of irrigation solution was used in the study groups.

Exposure of the teeth utilized in the experiment to the dry environment of in vitro studies causes dehydration, which may alter their mechanical properties [42]. Hence, researchers use many different liquids, such as artificial saliva and distilled water, HBSS, 0.9% NaCl, PBS, 0.2% thymol, and normal saline, to prevent the occurrence of dehydration. A study demonstrated a dramatic reduction in the hardness values of teeth maintained in NaCl for 30 days [43]. Considering the radiotherapy-induced alterations in the flow and content of patients’ saliva, artificial saliva also cannot fully mimic natural saliva as a storage medium [44]. Hence, we opted for the preservation of the teeth in distilled water, as in previous studies [8,45].

According to the in vitro findings of Duruk et al., although not statistically significant, an increase in dentin microhardness was observed at a radiation dose of 50 Gy. Moreover, at different radiation doses across the superficial, middle, and deep dentin layers, dentin hardness values exhibited both increases and decreases [46]. In another study, the results demonstrated a statistically significant reduction in microhardness in the dentino-enamel junction (DEJ) region, along with a decrease in enamel, whereas no significant difference was detected in dentin [6]. Notably, Lu et al. reported divergent responses of enamel and dentin to irradiation in terms of their mineral and organic composition. An increased protein-to-mineral ratio was observed in enamel with increasing radiation doses, whereas a decrease in this ratio was detected in dentin, reflecting a greater loss of organic content [6].

Although quasi-static fracture strength testing is commonly used for evaluating the mechanical performance of restorative materials [47,48], it does not fully replicate the complex cyclic and multidirectional forces occurring in the oral environment. This aspect may influence the findings and can be regarded as a limitation of this study. However, fracture testing was performed on all samples at the same angle and velocity, keeping all other standardized variables constant. Future studies incorporating cyclic and multidirectional loading of teeth may improve the clinical relevance and generalizability of the findings.

The absence of quantitative validation of the periodontal ligament simulation layer thickness represents a limitation of this study. Future research should adopt precise digital measurement and standardized fabrication protocols to enhance the reliability and mechanical consistency of experimental models.

Several limitations should be considered when evaluating the findings of this study. First, as an in vitro investigation, this research cannot fully replicate the complex biological and mechanical conditions of the oral environment, including masticatory forces, temperature, and the presence of saliva. Second, mandibular anterior teeth were used in this study, limiting generalizability to other tooth types with more complex anatomy such as premolars or molars. Third, our radiotherapy protocol (70 Gy delivered in fractionated doses) simulates clinical regimens for head and neck cancer. However, individual patient responses might yield different outcomes in vivo. Fourth, the lack of a radiation-only group represents a limitation of this study, as it may limit a direct assessment of radiation-induced changes in dental tissues independent of endodontic access and irrigation procedures. Future studies should include longitudinal, multicenter, and in vivo research to validate these findings and enhance potential applicability to larger populations. Clinical studies examining outcomes of patients would provide valuable insights into the long-term effects of radiotherapy and sonication on endodontically treated teeth.

## 5. Conclusions

Within the specific conditions of this in vitro protocol, neither radiotherapy nor sonic activation during irrigation significantly affected fracture resistance in endodontically treated teeth. However, root canal treatment itself significantly reduced the fracture resistance of mandibular anterior teeth compared to untreated controls. These preclinical findings provide a basis for future laboratory and potential clinical investigations.

## Figures and Tables

**Table 1:** Experimental design and treatment protocols for the five study groups. N: Number of sample size, cpm: cycles per minute.

Groups	N	Radiotherapy	Root Canal Treatment	Sonication and Irrigation Method	Obturation
Group I	16	None	None	None	None
Group II	16	None	Yes (35.04)	10 mL 2.5% NaOCl conventional irrigation without sonic activation (1 min)	Yes
Group III	16	None	Yes (35.04)	10 mL 2.5% NaOCl with EndoActivator sonic activation (10,000 cpm for 1 min)	Yes
Group IV	16	Yes (70 Gy)	Yes (35.04)	10 mL 2.5% NaOCl with EndoActivator sonic activation (10,000 cpm for 1 min)	Yes
Group V	16	Yes (70 Gy)	Yes (35.04)	10 mL 2.5% NaOCl conventional irrigation without sonic activation (1 min)	Yes

**Table 2:** Baseline buccolingual (BL) and mesiodistal (MD) root dimensions (mm) in the experimental groups (Mean ± SD).

Groups	BL Dimensions	MD Dimensions
Group I	6.72 ± 0.31 ^A^	4.14 ± 0.21 ^A^
Group II	6.81 ± 0.33 ^A^	4.13 ± 0.24 ^A^
Group III	6.74 ± 0.33 ^A^	4.15 ± 0.22 ^A^
Group IV	6.77 ± 0.32 ^A^	4.18 ± 0.27 ^A^
Group V	6.72 ± 0.31 ^A^	4.11 ± 0.20 ^A^

Values are presented as mean ± standard deviation. Same superscript letters indicate no statistically significant differences among groups (one-way ANOVA, *p* > 0.05).

## Data Availability

The raw data supporting the conclusions of this article will be made available by the authors on request.

## Abbreviations

The following abbreviations are used in this manuscript:

Gy	Gray (unit of radiation dose)
N	Newton (unit of force)
SEM	Scanning electron microscopy
kHz	Kilohertz
mm	Millimeter
n	Sample size
NaOCl	Sodium hypochlorite
mL	Milliliter
cpm	Cycles per minute
EDTA	Ethylenediaminetetraacetic acid
SD	Standard deviation
NS	Non-significant
